# Supplementary material for: Improved Fluorescent Proteins for Dual-Colour Post-Embedding CLEM
Source: Cells. 2022 Mar 23;11(7):1077. doi: 10.3390/cells11071077 (PMC8997867; doi:10.3390/cells11071077)
Supplement: Supplementary file 1 [file cells-11-01077-s001.zip › cells-1620367-supplementary.pdf]

Improved Fluorescent Proteins for Dual-Colour Post-Embedding CLEM

Dingming Peng [1,2,†], Na Li [1,3,4,5,†], Wenting He [1], Kim Ryun Drasbek [4,5], Tao Xu [2,6,*], Mingshu Zhang [1,2,*] and Pingyong Xu [1,2,6,7,*]

[1] Key Laboratory of RNA Biology, Institute of Biophysics, Chinese Academy of Sciences, Beijing, 100101, China; dmpeng7@gmail.com (D.P.); nali9415001@gmail.com (N.L.); hewenting@ibp.ac.cn (W.H.)

[2] Bioland Laboratory (Guangzhou Regenerative Medicine and Health Guangdong Laboratory),

Guangzhou 510005, China

[3] Sino-Danish College, University of Chinese Academy of Sciences, Beijing, 100101, China

[4] Sino-Danish Center for Education and Research, Beijing, 100039, China; ryun@cfin.au.dk

[5] Center of Functionally Integrative Neuroscience, Department of Clinical Medicine, Aarhus University, Aarhus, 8000, Denmark

[6] National Laboratory of Biomacromolecules, CAS Center for Excellence in Biomacromolecules, Institute of Biophysics, Chinese Academy of Sciences, Beijing, 100101, China

[7] College of Life Sciences, University of Chinese Academy of Sciences, Beijing, 101408, China

* Correspondence: xutao@ibp.ac.cn (T.X.); zhangmingshu@ibp.ac.cn (M.Z.); pyxu@ibp.ac.cn (P.X.)

† These authors contributed equally to this work.

**Figure S1**	Autofluorescence of an Epon resin slice
**Figure S2**	Green fluorescence emission properties of RFPs
**Figure S3**	Photoswitching properties of mEosEM and mutants before EM sample preparation
**Figure S4**	Scheme of sLM imaging of an EM sample
**Figure S5**	SBR comparison of mEosEM and mEosEM-E in OFF- and ON-images
**Figure S6**	Pre-fixation influence on RFPs
**Figure S7**	Fluorescence intensity of RFPs in pre-fixed cells followed by dehydration
**Figure S8**	SBR comparison of RFPs after EM sample preparation
**Figure S9**	Dual-color post-Epon-embedding CLEM imaging of Nopp52 and Nopp140
**Table S1**	Statistics for contrast ratio of mEosEM and mutants before EM sample preparation
**Table S2**	Statistics for contrast ratio comparison between mEosEM and mEosEM-E
**Table S3**	Statistics for SBR of mEosEM in OFF, ON, and ON－OFF images
**Table S4**	Statistics for SBR of mEosEM-E in OFF, ON, and ON－OFF images
**Table S5**	Statistics for SBR comparison between mEosEM and mEosEM-E in ON－OFF images
**Table S6**	Statistics for SBR comparison between mEosEM and mEosEM-E in OFF images
**Table S7**	Statistics for SBR comparison between mEosEM and mEosEM-E in ON images
**Table S8**	Statistics for fluorescence intensity comparison of RFPs in live cells
**Table S9**	Statistics for fluorescence intensity comparison of RFPs after pre-fixation
**Table S10**	Statistics for fluorescence intensity comparison of RFPs after 1% OsO_4 post-fixation
**Table S11**	Statistics for fluorescence intensity comparison of RFPs after 1% OsO_4 post-fixation and dehydration treatment
**Table S12**	Statistics for fluorescence intensity comparison of RFPs after dehydration treatment

**Table S13**	Statistics for thermostability comparison of RFPs
**Table S14**	Statistics for fluorescence intensity comparison of RFPs after EM sample preparation
**Table S15**	Statistics for SBR comparison of RFPs after EM sample preparation
**Table S16**	Statistics for photostability comparison of RFPs

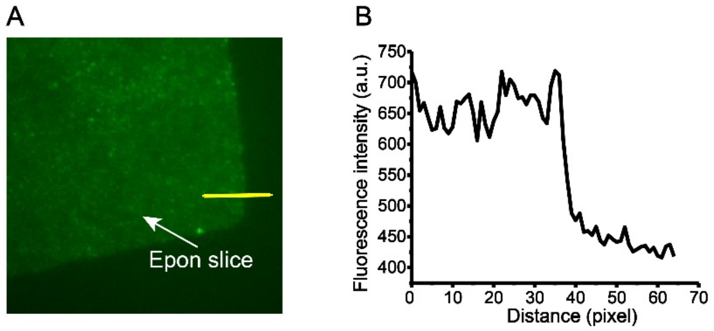

Figure S1. Autofluorescence of an Epon resin slice. (**A**) A corner of an Epon slice was imaged under 488-nm laser (0.41 kW/cm^2). (**B**) Fluorescence intensity profile of the yellow line in (**A**). Fluorescence intensity was plotted against distance.

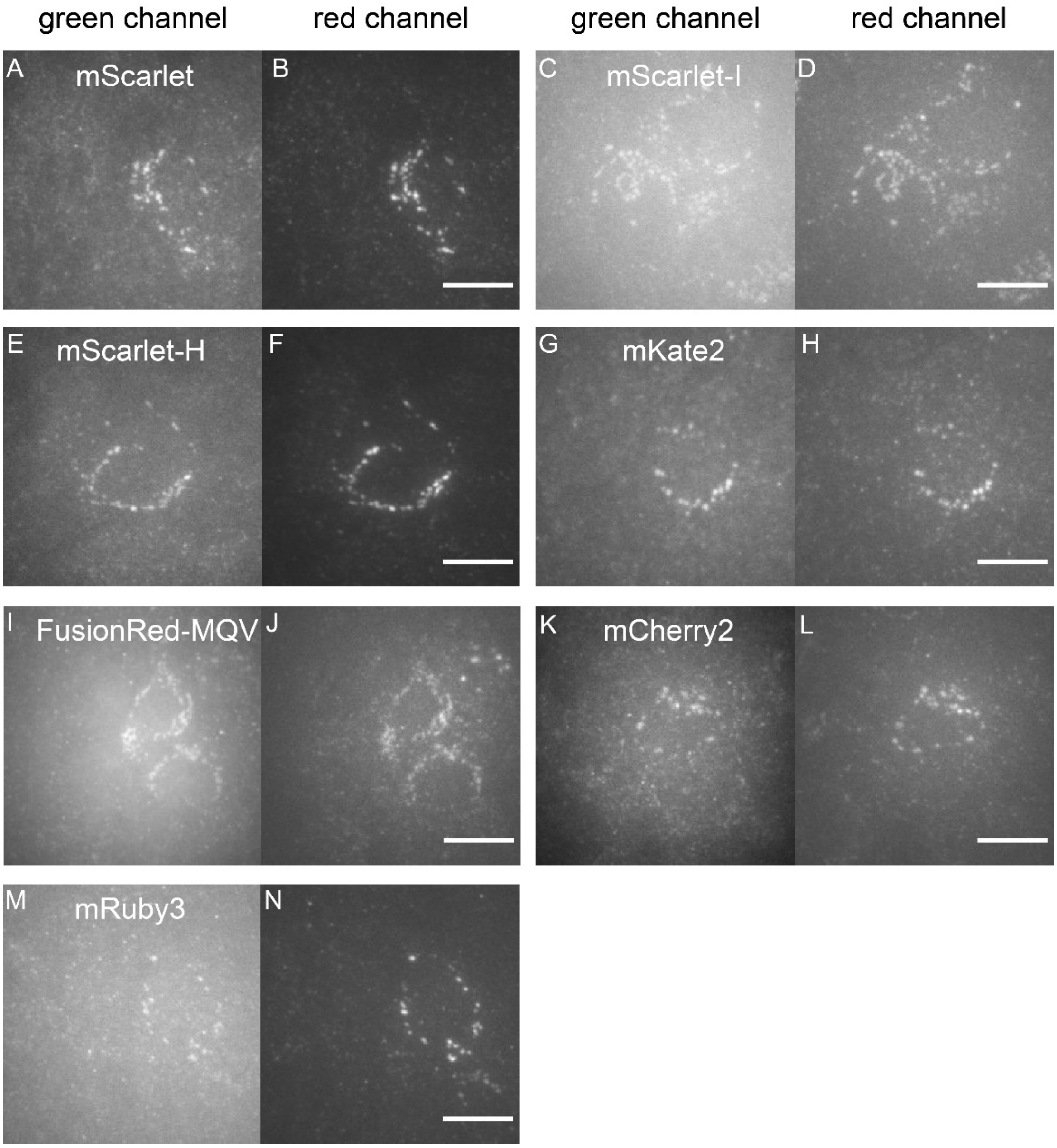

Figure S2. Green fluorescence emission properties of RFPs. HEK 293T cells expressing RFPs were prepared under standard EM sample preparation and sectioned into 100 nm slices. Sample slices were imaged by wide field fluorescence microscopy with sequential illumination of 561- and 488-nm laser. (**A**), (**C**), (**E**), (**G**), (**I**), (**K**) and (**M**) Slices were illuminated under 488-nm laser (0.92 kW/cm^2) and signals were recorded from the green channel. (**B**), (**D**), (**F**), (**H**), (**J**), (**L**) and (**N**) Slices were illuminated under 561-nm laser (0.57 kW/cm^2) and signals were recorded from the red channel. Scale bars, 10 μm.

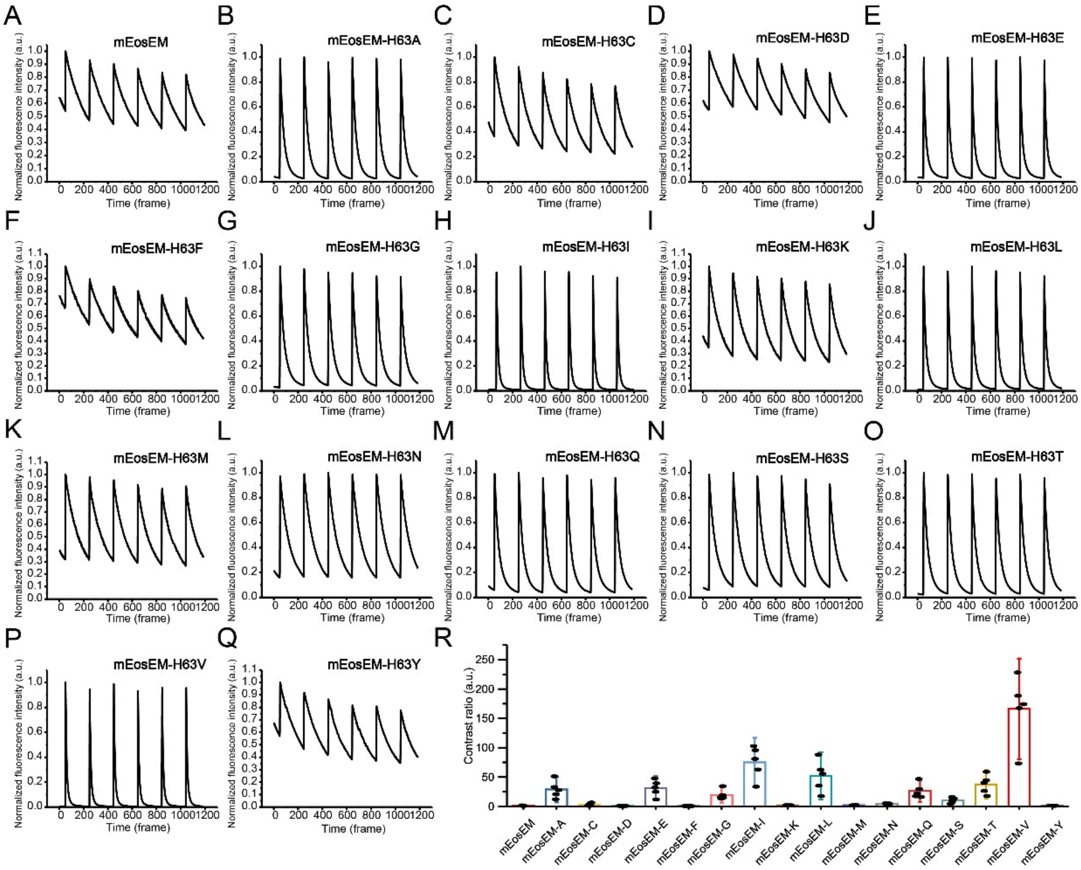

Figure S3. Photoswitching properties of mEosEM and mutants before EM sample preparation. (**A-Q**) Normalized fluorescence intensities of mEosEM and mutants were plotted against time. Cells expressing different fluorescent proteins were continuously illuminated with a 488-nm laser (8 W/cm^2), while every 10 s, a 405-nm laser pulse (0.1 s, 9 W/cm^2) were applied to switch on the FPs. Exposure time, 50 ms. (**R**) Mean contrast ratios of mEosEM and mutants before EM sample preparation. Error bars represent standard errors. n = 5. Data are summarized in Table S1.

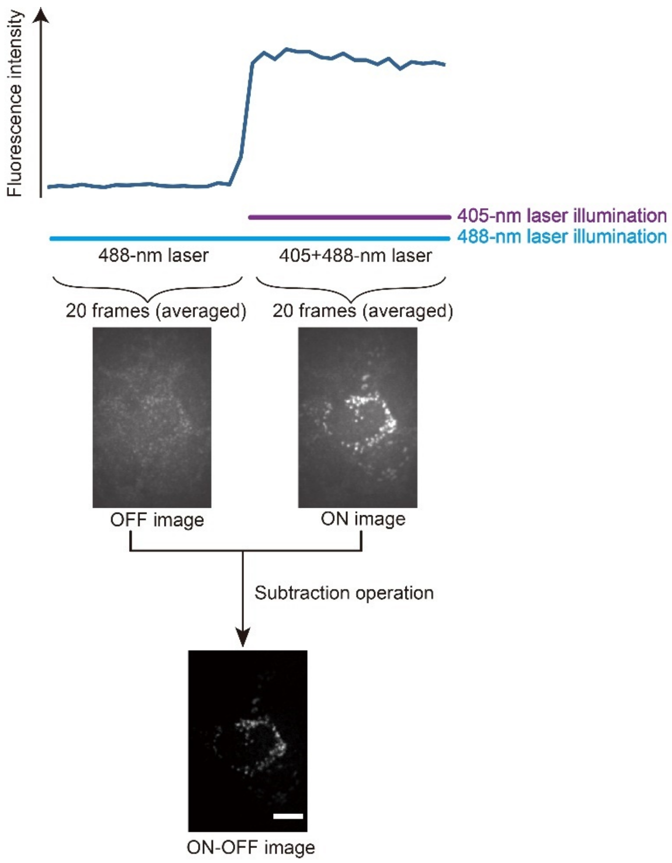

Figure S4. Scheme of sLM imaging of an EM sample. 100-nm cell sections were imaged under a continuous 488-nm laser for 50 frames, after which the 405-nm laser was added for 20 frames to record the fluorescence signal of the FPs during the on-state. The 20 frames immediately before 405-nm illumination were averaged to produce OFF images, and the subsequent 20 frames with 405-nm illumination were averaged to produce ON images. The sLM images (ON－OFF images) were generated by pixel-by-pixel subtraction using adjacent ON and OFF images. Scale bar, 5μm.

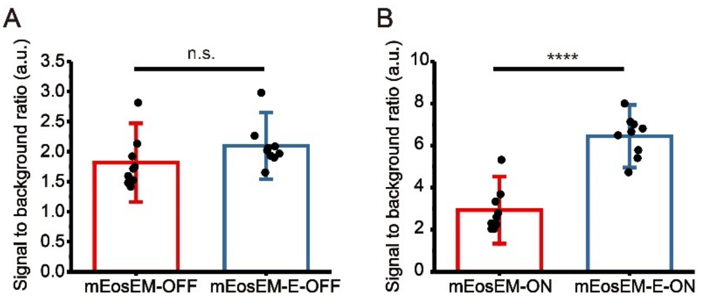

Figure S5. SBR comparison of mEosEM and mEosEM-E in OFF- and ON-images. (**A**) Statistics of SBR in OFF-images between mEosEM and mEosEM-E. (**B**) Statistics of SBR in ON-images between mEosEM and mEosEM-E. Bars represent mean ± SD. P-value were determined with two-tailed t-test in (**A-B**) (n = 9). n.s. indicates $p>0.05$, **** indicates $p<0.0001$. Data are summarized in Table S6 & S7.

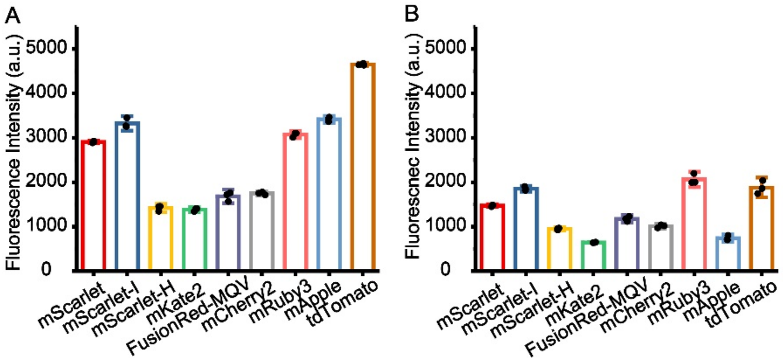

Figure S6. Pre-fixation influence on RFPs. (**A**) Fluorescence intensity of RFPs in live cells. Cells expressing RFPs were seeded in 96-well plates and imaged. (**B**) Fluorescence intensity of RFPs in pre-fixed cells. Cells expressing RFPs were seeded in 96-well plates and treated with fixation buffer (2.5% Glutaraldehyde and 2% Paraformaldehyde in PBS) for 15 min then washed 3 times with PBS buffer. Data were recorded using a high-content screening system. Excitation wavelength, 561 nm. Exposure time, 40 ms. Bars represent mean ± SD (n = 3). Data are summarized in Table S8 & S9.

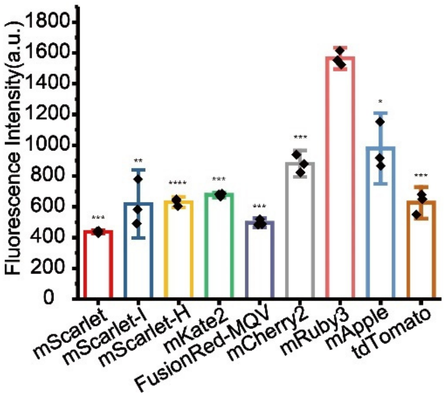

Figure S7. Fluorescence intensity of RFPs in pre-fixed cells followed by dehydration. Cells expressing RFPs were seeded in 96-well plates and fixed with fixation buffer (2.5% Glutaraldehyde and 2% Paraformaldehyde in PBS buffer) for 15 min, and then treated with absolute ethanol for 20 min. Data were recorded using a high-content screening system. Excitation wavelength, 561 nm. Exposure time, 40 ms. Bars represent mean ± SD. P-value were determined with two-tailed t-tests (n = 3). * indicates p<0.05. ** indicates p<0.01. *** indicates p<0.001. **** indicates p<0.0001. Data are summarized in Table S12.

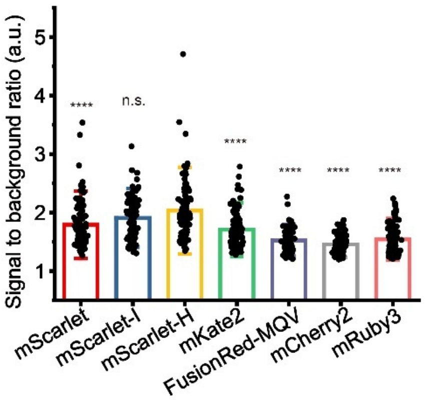

Figure S8. SBR comparison of RFPs after EM sample preparation. HEK 293T cells expressing RFPs were prepared under standard EM sample preparation and sectioned into 100 nm slices. Mann-Whitney U test was performed ($n = 106$). n.s. indicates $p > 0.05$, **** indicates $p < 0.0001$. Data are summarized in Table S15.

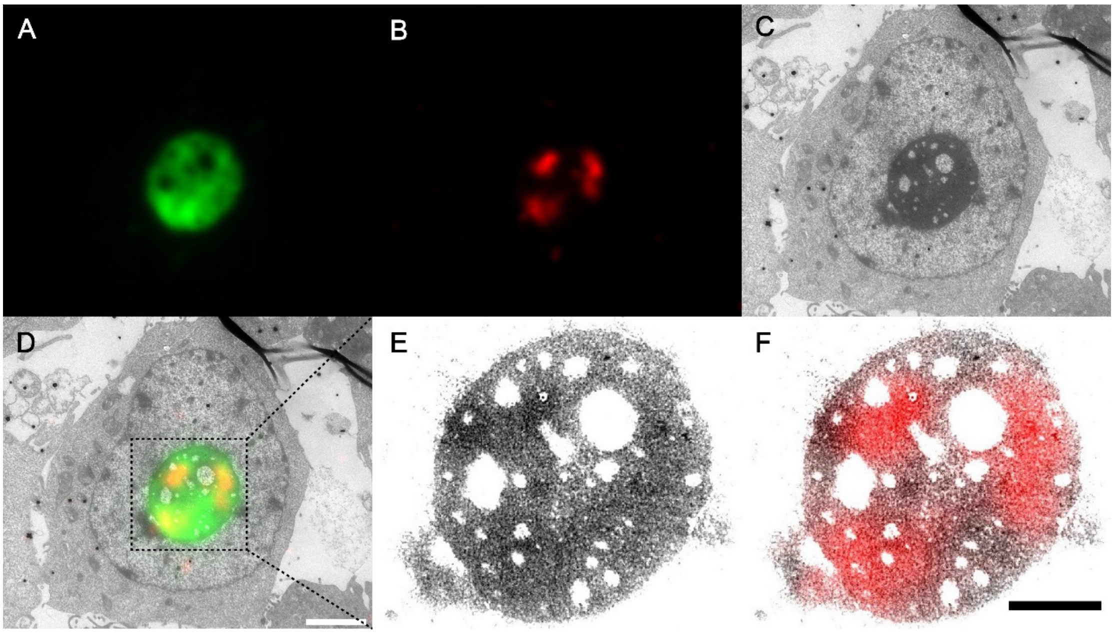

Figure S9. Dual-color post-Epon-embedding CLEM imaging of Nopp52 and Nopp140. (**A-D**) Dual-color CLEM imaging of mEosEM-E labeled Nopp52 and mScarlet-H labeled Nopp140 protein in HEK 293T cell sections (100 nm). Green channel FM (**A**), red channel FM (**B**), EM (**C**), and CLEM (**D**) images. Scale bar, 2 µm. (**E-F**) Enlarged EM (**E**) and red channel CLEM (**F**) images of boxed area in (**D**). Gamma value: 1.6 for both channels. Scale bar, 1 µm.

	Contrast ratio					mean	s.d.
mEosEM	0.623	1.114	0.845	0.821	1.673	1.015	0.407
mEosEM-A	20.716	32.975	12.130	51.253	27.805	28.976	14.711
mEosEM-C	0.840	1.117	4.340	6.428	1.744	2.894	2.412
mEosEM-D	0.758	0.814	0.408	0.751	0.577	0.662	0.167
mEosEM-E	39.382	32.132	47.701	24.849	11.781	31.169	13.762
mEosEM-F	1.428	0.626	0.691	0.505	0.488	0.747	0.389
mEosEM-G	15.291	19.369	14.976	34.559	13.255	19.490	8.718
mEosEM-I	102.815	62.832	81.008	33.619	95.741	75.203	27.840
mEosEM-K	2.102	1.887	1.420	1.964	1.854	1.845	0.256
mEosEM-L	62.654	17.426	88.161	55.673	35.340	51.851	26.960
mEosEM-M	1.926	1.776	2.145	2.118	2.663	2.126	0.336
mEosEM-N	3.957	4.793	4.574	2.872	5.173	4.274	0.899
mEosEM-Q	22.014	29.660	16.040	46.654	17.260	26.326	12.559
mEosEM-S	4.546	8.633	13.426	10.471	15.886	10.592	4.372
mEosEM-T	40.195	44.538	58.869	17.686	26.349	37.527	16.050
mEosEM-V	228.326	73.234	174.318	188.631	167.465	166.395	57.168
mEosEM-Y	0.757	1.103	1.248	1.331	1.028	1.093	0.223

Table S1. Statistics for contrast ratio of mEosEM and mutants before EM sample preparation. n = 5.

	mEosEM	mEosEM-E
Contrast Ratio	2.147	4.660
	2.513	7.366
	2.216	7.561
	2.256	7.351
	2.132	4.959
	2.233	4.190
	2.404	4.832
	2.289	6.791
	1.597	6.082
mean	2.199	5.977
s.d.	0.256	1.336
p value	2.13×10^{-5}	

Table S2. Statistics for contrast ratio comparison between mEosEM and mEosEM-E. Two-tailed t-test was performed between mEosEM and mEosEM-E, n = 9.

	mEosEM (OFF)	mEosEM (ON)	mEosEM (ON-OFF)
Signal-to-Background Ratio	2.130	3.684	12.289
	2.812	5.321	12.680
	1.921	3.336	11.894
	1.716	2.604	6.375
	1.416	2.035	5.226
	1.746	2.792	9.019
	1.478	2.304	9.097
	1.524	2.251	6.370
	1.593	2.039	3.676
mean	1.815	2.930	8.514
s.d.	0.437	1.062	3.298
p value			0.000762

Table S3. Statistics for SBR of mEosEM in OFF, ON, and ON − OFF images. Two-tailed t-test was performed between mEosEM (ON) and mEosEM (ON − OFF), n = 9.

	mEosEM-E (OFF)	mEosEM-E (ON)	mEosEM-E (ON-OFF)
Signal-to-Background Ratio	2.086	5.785	28.925
	1.651	4.731	16.408
	1.931	7.015	34.881
	2.020	7.132	27.124
	1.899	5.406	38.488
	2.978	8.000	22.849
	2.263	6.488	28.004
	1.966	6.808	37.050
	2.061	6.655	31.652
mean	2.095	6.447	29.487
s.d.	0.370	0.990	7.029
p value			7.87×10^{-6}

Table S4. Statistics for SBR of mEosEM-E in OFF, ON, and ON — OFF images. Two-tailed t-test was performed between mEosEM-E (ON) and mEosEM-E (ON — OFF), n = 9.

	mEosEM	mEosEM-E
Signal-to-Background Ratio	12.289	28.925
	12.680	16.408
	11.894	34.881
	6.375	27.124
	5.226	38.488
	9.019	22.849
	9.097	28.004
	6.370	37.050
	3.676	31.652
mean	8.514	29.487
s.d.	3.298	7.029
p value	4.70×10^{-6}	

Table S5. Statistics for SBR comparison between mEosEM and mEosEM-E in ON－OFF images. Two-tailed t-test was performed between mEosEM and mEosEM-E, n = 9.

	mEosEM-OFF	mEosEM-E-OFF
Contrast Ratio	2.130	2.086
	2.812	1.651
	1.921	1.931
	1.716	2.020
	1.416	1.899
	1.746	2.978
	1.478	2.263
	1.524	1.966
	1.593	2.061
mean	1.815	2.095
s.d.	0.436	0.370
p value	0.162	

Table S6. Statistics for SBR comparison between mEosEM and mEosEM-E in OFF images. Two-tailed t-test was performed between mEosEM and mEosEM-E, n = 9.

	mEosEM-ON	mEosEM-E-ON
Contrast Ratio	3.684	5.785
	5.321	4.731
	3.336	7.015
	2.604	7.132
	2.035	5.406
	2.792	8.000
	2.304	6.488
	2.251	6.808
	2.039	6.655
mean	2.930	6.447
s.d.	1.062	0.990
p value	1.94×10^{-6}	

Table S7. Statistics for SBR comparison between mEosEM and mEosEM-E in ON images. Two-tailed t-test was performed between mEosEM and mEosEM-E, n = 9.

	mScarlet	mScarlet-I	mScarlet-H	mKate2	FusionRed-MQV	mCherry2	mRuby3	mApple	tdTomato
Fluorescence									
Intensity	2928	3256	1453	1406	1568	1752	3011	3364	4674
	2906	3269	1457	1344	1763	1715	3100	3423	4637
	2887	3449	1348	1401	1719	1781	3104	3465	4643
Mean	2907	3324.667	1419.333	1383.667	1683.333	1749.333	3071.667	3417.333	4651.333
s.d.	20.51828	107.8718	61.80885	34.44319	102.2758	33.08071	52.57693	50.73789	19.85783

Table S8. Statistics for fluorescence intensity comparison of RFPs in live cells. n = 3.

	mScarlet	mScarlet-I	mScarlet-H	mKate2	FusionRed-MQV	mCherry2	mRuby3	mApple	tdTomato
Fluorescence									
Intensity	1490	1819	955.6	651.3	1117	974.2	1995	703.9	1860
	1483	1903	966.4	639.8	1228	1019	2198	715.2	2041
	1450	1841	924.8	642.8	1178	1041	2000	803.6	1745
Mean	1474.333	1854.333	948.9333	644.6333	1174.333	1011.4	2064.333	740.9	1882
s.d.	21.36196	43.55839	21.58642	5.965177	55.59077	34.04233	115.7857	54.59295	149.2213

Table S9. Statistics for fluorescence intensity comparison of RFPs after pre-fixation. n = 3.

	mScarlet	mScarlet-I	mScarlet-H	mKate2	FusionRed-MQV	mCherry2	mRuby3	mApple	tdTomato
Fluorescence									
Intensity	741.800	791.900	609.200	322.500	445.800	552.600	743.900	311.400	482.700
	712.100	764.000	592.400	320.800	440.100	505.400	738.000	304.400	495.900
	724.400	833.300	625.800	330.300	438.100	519.300	776.600	310.600	477.000
Mean	726.100	796.400	609.133	324.533	441.333	525.767	752.833	308.800	485.200
s.d.	14.923	34.868	16.700	5.066	3.995	24.255	20.793	3.831	9.695
p value									
(compared to									
mKate2)	0.000127	0.00150	0.000478				0.000423		
p value									
(compared to									
mCherry2)	0.000714	0.000695	0.0108				0.000286		

Table S10. Statistics for fluorescence intensity comparison of RFPs after 1% OsO_4 post-fixation. Two-tailed t-tests were performed between mKate2 and mScarlet, mScarlet-I, mScarlet-H, mRuby3, respectively, n = 3. Two-tailed t-tests were performed between mCherry2 and mScarlet, mScarlet-I, mScarlet-H, mRuby3, respectively, n = 3.

	mScarlet	mScarlet-I	mScarlet-H	mKate2	FusionRed-MQV	mCherry2	mRuby3	mApple	tdTomato
Fluorescence									
Intensity	321.400	277.300	314.200	298.100	314.400	332.200	395.900	317.100	325.000
	296.800	281.100	319.100	297.800	272.400	308.000	385.100	312.300	325.200
	333.600	285.700	309.500	305.300	283.900	297.300	407.500	309.300	295.700
Mean	317.267	281.367	314.267	300.400	290.233	312.500	396.167	312.900	315.300
s.d.	18.745	4.206	4.800	4.246	21.704	17.880	11.202	3.934	16.974
p value	0.00637	0.00114	0.00216	0.00177	0.00492	0.00435		0.00282	0.00388

Table S11. Statistics for fluorescence intensity comparison of RFPs after 1% OsO_4 post-fixation and dehydration treatment. Two-tailed t-tests were performed between mRuby3 and other RFPs, n = 3.

	mScarlet	mScarlet-I	mScarlet-H	mKate2	FusionRed-MQV	mCherry2	mRuby3	mApple	tdTomato
Fluorescence									
Intensity	429.100	583.500	648.900	685.600	518.800	938.800	1553.000	1153.000	680.100
	442.600	491.500	604.900	681.800	487.400	878.500	1615.000	917.600	650.000
	440.500	780.200	639.500	665.700	483.300	823.900	1525.000	865.900	549.900
Mean	437.400	618.400	631.100	677.700	496.500	880.400	1564.333	978.833	626.667
s.d.	7.264	147.480	23.172	10.565	19.421	57.474	46.058	153.032	68.164
p value	0.000420	0.00461	8.06×10^{-5}	0.000526	0.000102	0.000119		0.0157	0.000101

Table S12. Statistics for fluorescence intensity comparison of RFPs after dehydration treatment. Two-tailed t-tests were performed between mRuby3 and other RFPs, n = 3.

	mScarlet	mScarlet-I	mScarlet-H	mKate2	FusionRed-MQV	mCherry2	mRuby3	mApple	tdTomato
Thermostability									
Ratio	0.840	0.682	0.994	0.071	0.022	0.214	0.852	0.550	0.794
	0.842	0.742	0.959	0.071	0.021	0.206	0.856	0.575	0.728
	0.856	0.750	0.975	0.066	0.021	0.205	0.822	0.603	0.761
Mean	0.846	0.725	0.976	0.069	0.021	0.208	0.843	0.576	0.761
s.d.	0.009	0.037	0.017	0.003	0.001	0.005	0.018	0.026	0.033
p value	0.00151	0.00223		8.67×10^{-5}	0.000109	5.71×10^{-5}	0.000840	7.76×10^{-5}	0.00199

Table S13. Statistics for thermostability comparison of RFPs. Two-tailed t-tests were performed between mScarlet-H and other RFPs, n = 3.

	mScarlet	mScarlet-I	mScarlet-H	mKate2	FusionRed-MQV	mCherry2	mRuby3
Mean	238.979	283.083	327.015	210.963	153.836	136.175	158.454
s.d.	135.404	123.250	178.850	107.039	58.967	48.306	77.657
p value	0.000005	0.144		1.78×10^{-9}	3.23×10^{-21}	1.89×10^{-25}	1.19×10^{-18}

Table S14. Statistics for fluorescence intensity comparison of RFPs after EM sample preparation. Mann-Whitney U tests were performed between mScarlet-H and each RFP, n = 106.

	mScarlet	mScarlet-I	mScarlet-H	mKate2	FusionRed-MQV	mCherry2	mRuby3
Mean	1.791	1.910	2.034	1.706	1.524	1.456	1.541
s.d.	0.385	0.335	0.495	0.307	0.181	0.147	0.237
p value	0.000009	0.182		6.14×10^{-9}	8.95×10^{-22}	6.34×10^{-27}	1.64×10^{-18}

Table S15. Statistics for SBR comparison of RFPs after EM sample preparation. Mann-Whitney U tests were performed between mScarlet-H and each RFP, n = 106.

	mScarlet	mScarlet-I	mScarlet-H	mKate2	FusionRed-MQV	mCherry2	mRuby3
t1/e	341.040	359.090	476.280	366.780	269.560	307.030	636.990
	377.370	389.060	389.790	424.040	345.240	307.270	471.980
	351.770	353.860	452.630	426.160	273.080	327.990	527.020
	300.120	310.990	436.420	371.070	275.120	389.660	521.720
	380.550	433.362	440.220	290.670	253.760	381.610	482.540
Mean	344.147	366.071	443.090	362.633	267.320	366.420	510.427
s.d.	32.610	45.379	31.644	55.259	35.600	40.197	65.429
p value	0.0470	0.162		0.176	6.37×10^{-5}	0.0508	0.0299

Table S16. Statistics for photostability comparison of RFPs. Two-tailed t-tests were performed between mScarlet-H and other RFPs, n = 3.
